# The Effect of Cellular Differentiation on HSV-1 Infection of Oligodendrocytic Cells

**DOI:** 10.1371/journal.pone.0089141

**Published:** 2014-02-13

**Authors:** Raquel Bello-Morales, Antonio Jesús Crespillo, Beatriz García, Luis Ángel Dorado, Beatriz Martín, Enrique Tabarés, Claude Krummenacher, Fernando de Castro, José Antonio López-Guerrero

**Affiliations:** 1 Universidad Autónoma de Madrid, Departamento de Biología Molecular, Edificio de Biología, Darwin 2, Cantoblanco, Madrid, Spain; 2 Centro de Biología Molecular Severo Ochoa, CSIC-UAM, Cantoblanco, Madrid, Spain; 3 Universidad Autónoma de Madrid, Facultad de Medicina, Madrid, Spain; 4 Department of Pathobiology, University of Pennsylvania School of Veterinary Medicine, Philadelphia Pennsylvania, United States of America; 5 Grupo de Neurobiología del Desarrollo-GNDe, Hospital Nacional de Parapléjicos, Toledo, Spain; University of Illinois at Chicago, United States of America

## Abstract

Herpes simplex type 1 (HSV-1) is a neurotropic virus that infects many types of cells. Previous studies have demonstrated that oligodendrocytic cells are highly susceptible to HSV-1 infection. Here we analysed HSV-1 infection of a human oligodendrocytic cell line, HOG, and oligodendrocyte precursor cells (OPCs) cultured under growth or differentiation conditions. In addition to cell susceptibility, the role of the major cell receptors for viral entry was assessed. Our results revealed that OPCs and HOG cells cultured under differentiation conditions became more susceptible to HSV-1. On the other hand, viral infection induced morphological changes corresponding to differentiated cells, suggesting that HSV-1 might be inducing cell differentiation. We also observed colocalization of HVEM and nectin-1 with viral particles, suggesting that these two major HSV-1 receptors are functional in HOG cells. Finally, electron microscopy assays indicated that HSV-1 may be also entering OLs by macropinocytosis depending on their differentiation stage. In addition, vesicles containing intracellular enveloped virions observed in differentiated cells point to an endocytic mechanism of virus entry. All these data are indicative of diverse entry pathways dependent on the maturation stage of OLs.

## Introduction

Several infectious agents, ranging from mycobacteria to retroviruses, have been proposed to be associated with demyelinating diseases such as Multiple Sclerosis (MS), in which oligodendrocytes (OLs), the myelin-forming cells in the central nervous system (CNS), may be the initial target for the pathogenic onset [1], [2], [3]. Of all studied organisms, members of the viral family *Herpesviridae* are among the most promising candidates [3], [4], [5], [6], [7], [8]. In addition to other herpesviruses (for example Epstein-Barr virus or human herpesvirus 6), herpes simplex virus type 1 (HSV-1), has been linked to the possible aetiology or development of several neurodegenerative diseases and virus-induced demyelination [9], [10], [11], [12]. Previous reports have shown that a human oligodendrocyte-derived cell line is highly susceptible to HSV-1 [13], and that the virus may play a role in triggering MS relapses during clinical acute attacks of MS, at least in the most frequent clinical presentation of the disease, the relapsing-remitting form. [14]. Besides neurodegenerative diseases, HSV-1 may also be involved in cognitive alterations in bipolar or schizophrenia dysfunctions [15].

Herpesviruses usually infect their hosts for life, after the initial infection of epithelial cells, the virions spread to neurons and establish latent infections in sensory ganglia [16]. In some cases, the virus spreads into the CNS to cause encephalitis or meningitis [17]. HSV-1 entry into a diverse range of cell types has been described [18]. The entry of HSV into various cell types follows a complex process [19], [20].

The initial attachment of HSV-1 to the cell surface is mediated by glycoproteins B (gB) and C (gC). This interaction with heparan sulfate proteoglycans (HSPGs) enables the binding of viral gD to one of its receptors on the host cell surface. This binding triggers conformational changes in gD that allow the activation of gH/gL, which in turn activate the fusion effector gB [21], [22]. Cellular proteins binding to HSV gB have also been identified but their roles in the entry process or in cell tropism remains unsolved [23], [24], [25]. Molecules derived from three structurally different groups have so far been described as gD receptors in the host, Herpes Virus Entry Mediator (HVEM), a member of the tumor necrosis factor receptor family, nectin-1 and −2 from the immunoglobulin superfamily and distinctive sites in heparan sulfate (HS) generated by a specific 3-O-sulfotransferase (3-O-ST) [26], [27], [28], [29]. Nectin-1 and HVEM appear to be the principal gD-binding entry receptors although they bind distinct regions of the gD ligand [20]. They are coexpressed in many cells and used by the majority of tested clinical strains of HSV-1, as well as HSV-2 [30]. HVEM expression has been found in liver, kidney, lymphoid tissues, lung and in several cell lines. Nectin-1 is the main, although not exclusive, HSV receptor on epithelial and neuronal cells, whereas nectin-2 use seems to be limited to only few viral mutant strains [27], [30], [31], [32], [33]. It is worth noting that nectin-1 is an adhesion molecule present at adherent junctions in polarized cells, such as epithelial and neurons cells, and in cell-cell contact in some cultured cells [34]. 3-O-ST HS can be used as an entry receptor for HSV-1 but not HSV-2 in multiple cell lines like neuronal or endothelial cells [27], [35]. Although in all cases, binding of gD to a specific receptor is required during HSV entry, membrane fusion can take place directly at the cell surface or, in some cases, following virus endocytosis. Why the virus chooses one or another pathway is largely unknown. However, studies with cell cultures of different origin –SY5Y, HeLa or Vero cell lines– suggest that nectin-1-mediated internalization may direct HSV to the endocytic pathway, possibly with the cooperation of integrins [36], [37], [38].

Finally, binding of HSV-1 to its cellular receptor –or receptors– seems to be sufficient for the induction of intracellular signalling even in the absence of subsequent virion entry [39]. Differential expression of cellular genes associated with NF-κB, Jak/Stat or p13K/Akt pathways has been observed by means of microarray studies, highlighting the effect of HSV-1 glycoproteins, particularly gD, on this process [39], [40].

Oligodendrocyte precursor cells (OPCs) give rise to oligodendrocytes during embryonic and postnatal development as well as in the adult CNS and can be differentiated *in vitro* into mature myelin-forming OLs [41], [42], [43], [44]. *In vitro*, OLs are characterized by a complex arborisation of cell processes and *in vivo*, these processes terminate in flat membranous sheets –rich in myelin proteins and lipids– that spirally wrap around and insulate neuron axons [45]. In the present report, we characterize HSV-1 infection of a human oligodendrocytic cell line, HOG, and OPCs in primary cell culture. Cells were cultured in growth or differentiation media, their differential susceptibility to viral infection was determined and the role of the major cell receptors for viral entry was investigated.

## Materials and Methods

### Antibodies and Reagents

Anti nectin-1 monoclonal antibody CK41 and anti-HVEM polyclonal antibody R140 have been described previously [46], [47]. Horseradish peroxidase-conjugated secondary anti-IgG antibodies were purchased from Millipore (Billerica, MA, USA). Anti-green fluorescent protein GFP rabbit polyclonal serum A6455, Alexa 488-, Alexa 647- and Alexa 594-conjugated secondary antibodies were obtained from Molecular Probes (Eugene, OR, USA). DNA size marker was from Invitrogen. Polyclonal rabbit anti-HSV-1 antibody was from DAKO. Monoclonal mouse anti-PLP MAB388 antibody was from Millipore. Anti-nectin-1 mouse monoclonal antibody CK6 was from Santa Cruz Biotechnology. Anti-HVEM mouse monoclonal antibody, low-glucose DMEM, fetal bovine serum (FBS), human insulin, triiodothyronine (T3), apo-transferrin, sodium selenite, putrescine, dibutyryl cyclic AMP (dbcAMP), carboxymethylcellulose sodium salt (CMC) medium-viscosity and protease inhibitor cocktail were purchased from Sigma Chemical Co. (St. Louis, MO, USA). Mowiol was from Calbiochem (Merck Chemicals, Germany). HS4C3 antibody was a kind gift of Dr. R. Longnecker, (Northwestern Medical School, Chicago, USA).

### Cells and Virus

The HOG cell line, established from a surgically removed human oligodendroglioma [48] was kindly provided by Dr. A. T. Campagnoni (University of California, UCLA, USA). Cells were cultured on Petri dishes in growth medium (GM) containing low-glucose DMEM supplemented with 10% fetal bovine serum (FBS), penicillin (50 U/mL) and streptomycin (50 µg/mL) at 37°C in an atmosphere of 5% CO2. To induce differentiation, cells were cultured in serum-free differentiation medium (DM) containing low-glucose DMEM supplemented with antibiotics and 50 µg/ml apo-transferrin, 0.5 mg/l insulin, 30 nM triiodothyronine (T3), 30 nM sodium selenite and 16.1 mg/l putrescine. Cells cultured in this medium were also treated with 0.5 mM dbcAMP and IBMX at a final concentration of 0.5 mM.

OPCs from postnatal P0 mice were generated as described [42], [49], [50] in the facilities of Hospital Nacional de Parapléjicos (Toledo, Spain). All animal experiments were carried out in accordance with Spanish (RD233/88) and European (2010/63/EU) regulations, and they were approved by the Animal Review Board at the Hospital Nacional de Parapléjicos (SAPA001). To differentiate OPCs, cells were maintained in differentiation medium [42] for 3 days. Cells cultured in that same medium for 24 h were considered as undifferentiated control.

K26GFP was a kind gift of Dr. Desai (Johns Hopkins University, Baltimore, USA). It was obtained by fusing GFP to the HSV-1 capsid protein VP26 [51]. The R120vGF, EGFP recombinant virus was propagated in E5 cells, a Vero cell line expressing the ICP4 protein of HSV-1 [52]. K26GFP and wild type HSV-1 (F strain, DNA genome sequence GenBank GU734771) viruses were propagated and titrated on Vero cells. GFP-MAL2/MAL-diHcRed/HOG cells areHOG cells stably transfected with GFP-MAL2,a construct encoding a chimera consisting of GFP fused to the amino-terminal end of MAL2, and with MAL-diHcRed, a construction consisting of MAL protein tagged with diHcRed, a dimeric red fluorescent protein [53].

### Viral Infections

For viral infection assays, 1.2x106 HOG cells growing in 25 cm2 flasks were mock-infected or infected with the corresponding virus. During viral adsorption, cells were maintained in DMEM with antibiotics in the absence of FCS. Subsequently, cultures were rinsed and cultured in its corresponding medium. Viral titer was quantified by an endpoint dilution assay determining the TCID50 in Vero cells, considering the final dilution that shows cytopathic effect and using the Reed and Muench method.

For plaque assay, confluent monolayers of cells plated in 6-well tissue culture dishes were infected with serial dilutions of HSV-1. After viral adsorption, cells were washed and overlaid with CMC. The CMC solution was prepared in distilled water at 2% (w/v) and stirred at room temperature for one hour. CMC overlay (1% final concentration) was prepared by mixing equal volumes of CMC 2% and 2x concentrated GM or DM. Two millilitres of CMC overlay were added to each well. Plates were incubated at 37°C in a humidified 5% CO2 incubator for 48 hours. The CMC overlay was then aspirated, cells were washed with PBS and fixed in 4% paraformaldehyde for 20 min. Plaques were visualized by staining with crystal violet.

### Construction and Characterization of R120vGF Recombinant HSV-1 Virus

**Figure 1:**
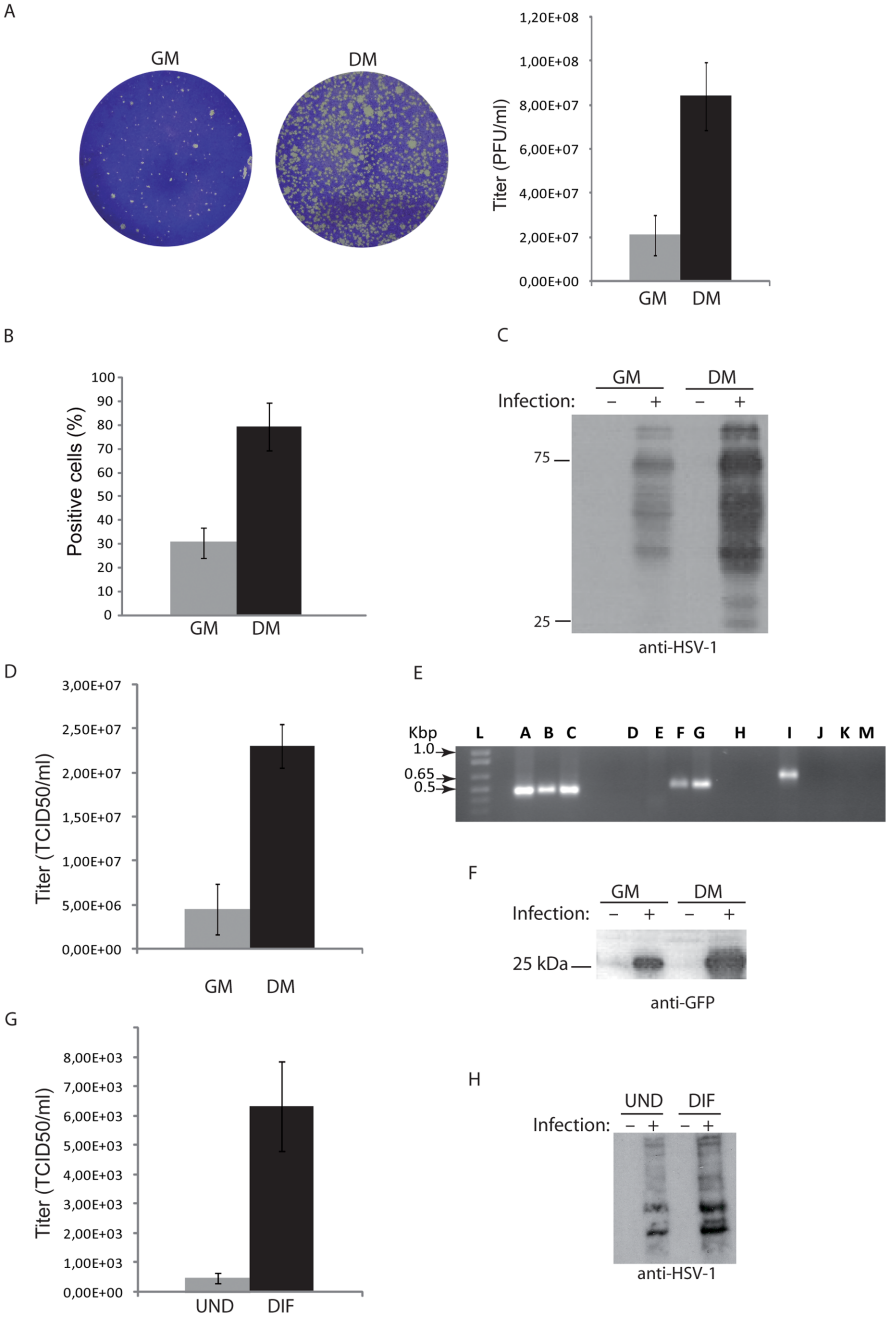
Effect of cell differentiation on HOG susceptibility to HSV-1 infection. A. Monolayers of HOG cells were infected with the same dose of HSV-1, overlaid with GM or DM containing CMC and stained with crystal violet. An increase in the number of plaque forming units (p.f.u.) per ml in differentiated cells compared to cells cultured in GM can be observed. The titration graph corresponds to the titration of a common stock on DM- and GM-cultured cells using the plaque assay. B. Cells mock-infected or infected at an m.o.i. of 0.5 with HSV-1 K26GFP were processed for flow cytometry analysis. The percentage of infection in differentiated cells is considerably higher than in cells cultured in GM. C. HOG cells cultured in GM or DM were mock-infected or infected with HSV-1 at an m.o.i. of 0.1, subjected to SDS–PAGE 24 h p.i. and analyzed by immunoblotting with a polyclonal rabbit anti-HSV-1 antibody. In cells cultured in DM, detection of viral proteins is increased. D. HOG cells cultured in GM or DM were infected with HSV-1 at an m.o.i. of 0.1. Viral titers at 20 h p.i. were determined by TCID_50_/ml. Virus yield was significantly superior in cells cultured in DM. E. Characterization of virus R120vGF. PCR amplification of the genes encoding thymidine kinase (TK) (lane A), virion host shutoff (*vhs*, UL41) (lane E) and chimeric EGFP (lane I) from R120vGF DNA. PCR amplification of the TK (lane B), *vhs* gene (lane F) and chimeric EGFP (lane J) from parental HSV-1 strain d120 DNA. PCR amplification of the genes encoding TK (lane C), *vhs* (lane G) and chimeric EGFP (lane K) from HSV-1 strain F DNA. Negative PCR controls without DNA (lanes D, H and M). PCR products were electrophoresed in 1% agarose gel. A 1 kb DNA ladder was used as a DNA size marker (lane L). F. Cells cultured in GM and DM and mock-infected or infected with R120vGF at an m.o.i. of 0.1 were subjected to SDS–PAGE 24 h p.i. and analyzed by immunoblotting with polyclonal anti-GFP antibody. In cells cultured in DM, the viral-associated GFP signal is significantly higher. Susceptibility to HSV-1 infection was also evaluated in undifferentiated (UND) or differentiated (DIF) OPCs. Both viral titer at 20 h p.i. determined by TCID_50_/ml (G) and immunoblot signal detected with a rabbit polyclonal anti-HSV-1 antibody at 20 h p.i (H) is higher in differentiated cells.

The recombinant R120vGF virus was obtained by transfecting plasmid DNA of pUH41GF digested with *EcoRI* and *HindIII* into E5 cells, infected with HSV-1 mutant strain d120 deficient in ICP4 [52], using lipofectamine 2000 (Invitrogen). The recombinant progeny was selected by using EGFP expression as a marker. Recombinant virus was plaque-purified five times in E5 cells. The amino terminal deletion of the *vhs* gene was confirmed by PCR characterization of viral DNA of R120vGF. This was carried out with primers HTK6D (sense) (5′-GCAAGAAGCCACGGAAGTCC-3′) and HTK6R (antisense) (5′- ATGAGGGCCACGAACGCCAG-3′) for the HSV-1 TK gene, HL41S (sense) (5′-ACAATTGACCTGCCATGG-3′) and HL41AS (antisense) (5′-CGAATACAGAACAGATGC-3′) for the HSV-1 UL41 (*vhs*) gene and p41HS (5′-TTGGAAGAGGCAATGAGC-3′) and GFP-AS (5′TAGGTCAGGGTGGTCACG-3′) for the chimeric EGFP gene of recombinant virus. PCR products were analyzed by 1% agarose gel electrophoresis, and the specificity of the amplification products was confirmed by DNA sizes of 479 bp for the HSV-1 TK (nt 102 to 581 of coding TK sequence), 540 for the UL41 (nt −13 to 527 from ATG of UL41) and 658 bp for the chimeric EGFP gene, respectively (Fig. 1E). The replacement of the EGFP cassette by the *SphI-EcoRV* UL41 fragment in R120vGF virus was confirmed because specific fragments from the TK and UL41 genes were amplified from DNA of parental HSV-1 strain d120 (Fig. 1E, lanes B and F) and DNA of HSV-1 strain F (Fig. 1E, lanes C and G). Using DNA of the R120vGF virus as template, specific fragments could be amplified from the TK gene by using HTK6D and HTK6R primers (Fig. 1E, lane A) but not from the UL41 gene by using HL41S and HL41AS primers (Fig. 1E, lane E), since this had been replaced by the EGFP chimeric gene amplified by p41HS and GFP-AS primers (Fig. 1E, lane I). DNA from infected cells was isolated by QIAamp DNA Micro Kit (QIAGEN).

### Antibody Blocking Assay

HOG cells cultured in a 24-well tissue culture dish were washed with free-serum DMEM and incubated with 10 µl of antibodies (1∶10 dilution) to block their corresponding receptor: R140 to block HVEM and CK41 to block nectin-1. Incubation with both antibodies simultaneously was also performed. Following incubation at 4°C for 1 h, an equal volume of K26GFP diluted in free serum medium was added to cells at an m.o.i of 1. Virus was incubated at 4°C for 1 h. After viral adsorption, cells were washed with PBS, incubated for 20 h with their respective media containing blocking antibodies and processed for flow cytometry. Cells not blocked with primary antibody were used as controls.

### Detection of Heparan Sulfates

To visualize HSPGs, we cultured HOG cells in GM or DM. After 24 hours, cells were washed with free-serum DMEM and incubated for 20 minutes at 4°C with WGA-594 (5 µg/ml). Then, cells were washed twice in PBS, fixed in 4% paraformaldehyde for 20 min and washed in PBS. Finally, cells were incubated with TO-PRO-3 to stain nuclei. To detect 3OS-HS we used HS4C3 antibody. HOG cells were cultured in GM or DM. After 24 hours, cells were fixed in 4% paraformaldehyde for 20 min, washed in PBS and permeabilized with 0.2% Triton X-100. After that, cells were blocked with 3% bovine serum albumin in PBS for 30 min and incubated with HS4C3 antibody (diluted 1∶10 in blocking solution) for 1 hr at room temperature. Both incubations were performed in the presence of 0.5 M NaCl to avoid unspecific crossreaction of the antibody.

### Immunoblot Analysis

Samples were subjected to SDS-PAGE in 10% acrylamide gels under reducing conditions and transferred to Immobilon-P membranes (Millipore). After blocking with 5% non-fat dry milk, 0.05% Tween 20 in PBS, blots were incubated for 1 h at room temperature with primary antibodies. After several washes with 0.05% Tween 20 in PBS, blots were incubated for 1 h with secondary antibodies coupled to horseradish peroxidase, washed extensively, and developed using an enhanced chemiluminescence Western blotting kit (ECL, Amersham, Little Chalfont, UK).

### Real-time Quantitative RT-PCR Assay

Real-time quantitative RT-PCR assay was performed as previously described [54]. Briefly, total RNA from triplicate samples of HOG cells infected with HSV-1 cultured in 60-mm dishes under growth or differentiation conditions was extracted using RNeasy Qiagene Mini kit (Qiagen, Valencia, CA, USA). RNA integrity was evaluated on Agilent 2100 Bioanalyzer (Agilent Technologies, Santa Clara, CA) and quantification of RNA was carried out in a Nanodrop ND-1000 spectrophotometer (Thermo Fisher Scientific). All the samples showed 260/280 ratio values around 2, which correspond to pure RNA. RNA Integrity Number (RIN) values were between 9.3 and 10, corresponding to RNA samples with high integrity. Genomic DNA contamination was assessed by amplification of representative samples without reverse transcriptase (RT). RT reactions were performed using the High Capacity RNA-to-cDNA Master Mix (Applied Biosystems PN 4390712) following manufacturer’s instructions. Primer sequences (5′–3′) were as follows: for nectin-1, ACTCGCTCTCGGCTTGAC and CCATACATGGAGTCGTTCACC; for HVEM, ATCCTGCTAGCTGGGTTCC and GGAAGGTGAGATACAGCACCA. We used the NormFinder algorithm to identified 18S as the most suitable genes for the normalization due to its high stability.

### Immunofluorescence Microscopy

Cells grown on glass coverslips were fixed in 4% paraformaldehyde for 20 min and rinsed with PBS. Cells were then permeabilized with 0.2% Triton X-100, rinsed and incubated for 30 min with 3% bovine serum albumin in PBS. For double and triple-labeled immunofluorescence analysis, cells were incubated for 1 h at room temperature with the appropriate primary antibodies, cells were then rinsed several times and incubated at room temperature for 30 min with the relevant fluorescent secondary antibodies. Controls to assess labeling specificity included omission of the primary antibodies. After thorough washing, coverslips were mounted in Mowiol. Images were obtained using an LSM510 META system (Carl Zeiss) coupled to an inverted Axiovert 200 microscope. Processing of confocal images and colocalization analysis was made by FIJI-ImageJ software.

### Flow Cytometry Analysis

To perform FACS analysis, HOG cells were dissociated in 0.05% trypsin/0.1% EDTA (Invitrogen) for 1 minute at room temperature, then washed and fixed in 4% paraformaldehyde for 15 minutes and, finally, rinsed and resuspended in PBS. Cells were analyzed using a FACSCalibur Flow Cytometer (BD Biosciences).

### Electron Microscopy

HOG cells cultured at 37°C in GM or DM were mock-infected or infected with HSV-1 at an m.o.i. of 50. At different time points post-infection, cells were fixed in 4% paraformaldehyde in 0.1 M sodium phosphate buffer, pH 7.4, at 37°C for 2 hours. Then, they were washed in PBS containing 20 mM glycine and processed by freeze substitution as previously described [13]. Samples were examined with a JEM 1010 transmission EM (Jeol, Tokyo, Japan).

## Results

### Culturing HOG Cells in Differentiation Medium Increases Susceptibility to HSV-1

The susceptibility of a human oligodendrocyte-derived cell line was previously assessed in our laboratory [13]. Here, we analyze the effect of oligodendrocytic differentiation on HSV-1 infection. HOG cells were cultured in GM or DM and infected with HSV-1. Plaque assay showed a significantly larger number of plaques in cells cultured in DM compared to cells cultured in GM when cells are infected with the same viral dose (Figure 1A). Similar results were obtained by flow cytometry analysis of HOG cells infected at an m.o.i. of 0.5 with GFP-tagged HSV-1 K26GFP. As shown in Figure 1B, a significant increase in the number of GFP-expressing cells 24 hours after infection was confirmed in cells cultured in DM compared to GM cultures. Immunoblotting assay also showed an increase in viral protein detection in cells cultured in DM compared to those cultured in GM (Figure 1C). To avoid differences in the number of cells in GM and DM cultures, we took into account the growth rate of GM and DM cells, so that at the time of infection, the number of cells in both cultures were the same. Although at 20 h p.i. the number of cells did not vary significantly, to control the amount of protein loaded, we performed the experiment either loading equal number of cells or equal amount of protein, obtaining similar results in both cases.

Finally, HOG cells were cultured in GM or DM and infected at an m.o.i of 0.1 with HSV-1. Progeny virus was titrated to determine the 50% tissue culture infective dose (TCID50)/ml. After 20 h p.i., viral yield in DM-cultured HOG cells was significantly higher compared to cells cultured in GM (Figure 1D). To investigate whether the increment in viral yield was due, at least in part, to an increase in viral entry, we carried out the infection of HOG cells using R120vGF, an EGFP-expressing recombinant HSV-1 lacking ICP4. Figure 1E shows PCR amplification of the genes encoding thymidine kinase (TK), virion host shutoff (*vhs*, UL41) and chimeric EGFP from R120vGF DNA (lanes A, E and I respectively); parental HSV-1 strain d120 DNA (lanes B, F and J respectively) and HSV-1 strain F DNA (lanes C, G and K respectively). Negative PCR controls without DNA are also shown (lanes D, H and M). After entry into cells, R120vGF expresses EGFP and immediate early proteins, but is unable to complete the viral cycle due to the absence of ICP4. Using this tool, we can measure GFP signal and immediate early protein production to estimate whether HSV entry is altered in HOG cells cultured under differentiation conditions. This novel viral construction allows to estimate HSV-1 entry determining either GFP fluorescence –by flow cytometry or immunofluorescence– or by immunoblot, providing new methods to the study of HSV-1 entry into cells. HOG cells cultured in GM and DM were infected with R120vGF at an m.o.i. of 0.1. After 24 h p.i., equal amounts of protein were subjected to SDS–PAGE and analyzed by immunoblotting with anti-GFP antibody. As in the previous experiments, an increase in viral signal was observed in HOG cells cultured in DM (Figure 1F), suggesting that differentiation is affecting viral entry. As indicated above, we observed an increase in the number of plaques in HOG cells cultured in DM compared to cells cultured in GM. However, the average size of plaques corresponding to cells cultured in DM was also increased, suggesting that other factors –apart from viral entry– might also be involved.

To extend the results obtained with HOG cells to primary cultures, we studied HSV-1 infection in mouse OPCs. Primary OPCs cultured in differentiation medium for 24 h (undifferentiated) or 3 days (to allow spontaneous differentiation) were infected at an m.o.i. of 1 with HSV-1, and the viral productivity was titrated 20 h p.i determining the TCID50/ml. Viral yield in differentiated cells was significantly higher compared to undifferentiated cells (Figure 1G). Also, immunoblotting assay showed an increase in viral protein detection in differentiated OPCs cultured for 3 days compared to undifferentiated cells cultured for 24 hours (Figure 1H).

### HSV-1 Infection Induces Differentiation in HOG Cells

**Figure 2:**
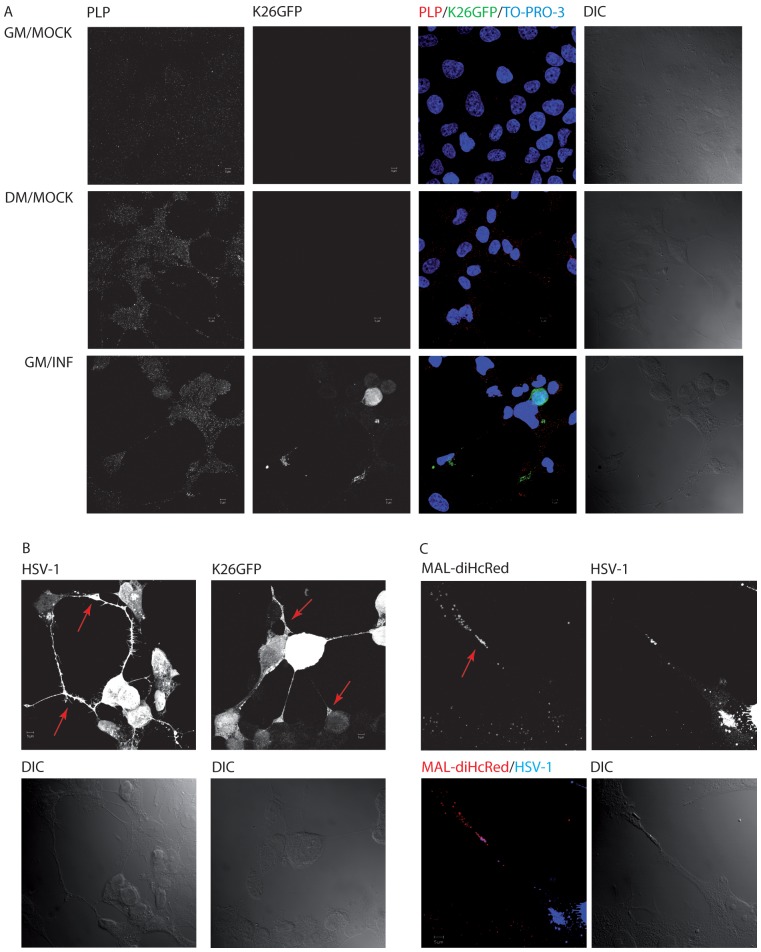
Effect of HSV-1 infection on cell differentiation of HOG cells. Cells cultured in GM or DM were mock-infected or infected at an m.o.i of 0.5 with K26GFP for 20 h. Then, cells were fixed and processed for confocal immunofluorescence analysis with an anti-PLP monoclonal antibody detected with an Alexa Fluor 555 secondary antibody. PLP signal is increased in mock-infected cells cultured in DM and in infected cells cultured in GM (A). In addition, membrane processes and myelin-like sheets (arrows) can be noticed in cells cultured in GM infected with HSV-1 or K26GFP (B). C. GFP-MAL2/MAL-diHcRed/HOG cells cultured in GM or DM were mock-infected or infected at an m.o.i of 0.5 with HSV-1 for 20 h. Cells were then fixed and processed for confocal immunofluorescence analysis with an anti-HSV-1 polyclonal antibody and an Alexa Fluor 647 secondary antibody. Myelin-like sheets enriched in exogenous MAL (arrow) can be observed in infected cells cultured in GM. All images correspond to the projection of the planes obtained by confocal microscopy. In panel A nuclei were stained with TO-PRO-3. DIC: Differential Interference Contrast. To make the cells more visible, DIC contrast of the whole images has been adjusted.

Once it was established that culturing HOG cells in differentiation medium increased infection by HSV-1, we decided to ascertain whether viral infection was also able to induce changes corresponding to a more advanced differentiation stage. For this purpose, HOG cells grown on glass coverslips were cultured in GM or DM and subsequently mock-infected or infected at an m.o.i. of 0.5 with K26GFP for 20 h. As previously observed [55], we detected an increase of proteolipid protein (PLP) levels in HOG cells cultured in DM (Figure 2A). Interestingly, an increase in PLP levels was also detected in cells cultured in GM and infected with K26GFP (Figure 2A). Surprisingly, PLP increased not only in infected cells, but also in non-infected cells. It is possible that contact with non-infectious particles or infected cells may be sufficient to trigger a response that induces differentiation of non-infected cells. Alternatively, factors secreted by infected cells might induce differentiation of non-infected cells. Further experiments will be needed to test these two possibilities. In addition, myelin-like sheets and other morphological features corresponding to differentiated cells were also observed in infected cells cultured in GM (Figure 2B). Finally, GFP-MAL2/MAL-diHcRed/HOG cells [53] grown on glass coverslips were cultured in GM or DM and thereafter mock-infected or infected at an m.o.i of 0.5 with HSV-1. Cells cultured in GM, exhibited myelin-like sheets enriched in exogenous MAL –a major myelin protein– (Figure 2C). All these data suggest that, even in growth medium, HSV-1 infection can induce a more differentiated stage in HOG cells. Finally, an unexpected partial colocalization of HSV-1 and exogenous MAL was observed (Figure 2C), especially in vesicles located at the end of the processes, suggesting that viral particles could be travelling into MAL-positive vesicles during viral egress. However, further studies will be necessary to demonstrate this hypothesis.

### Expression of HSV-1 Receptors in HOG Cells

**Figure 3:**
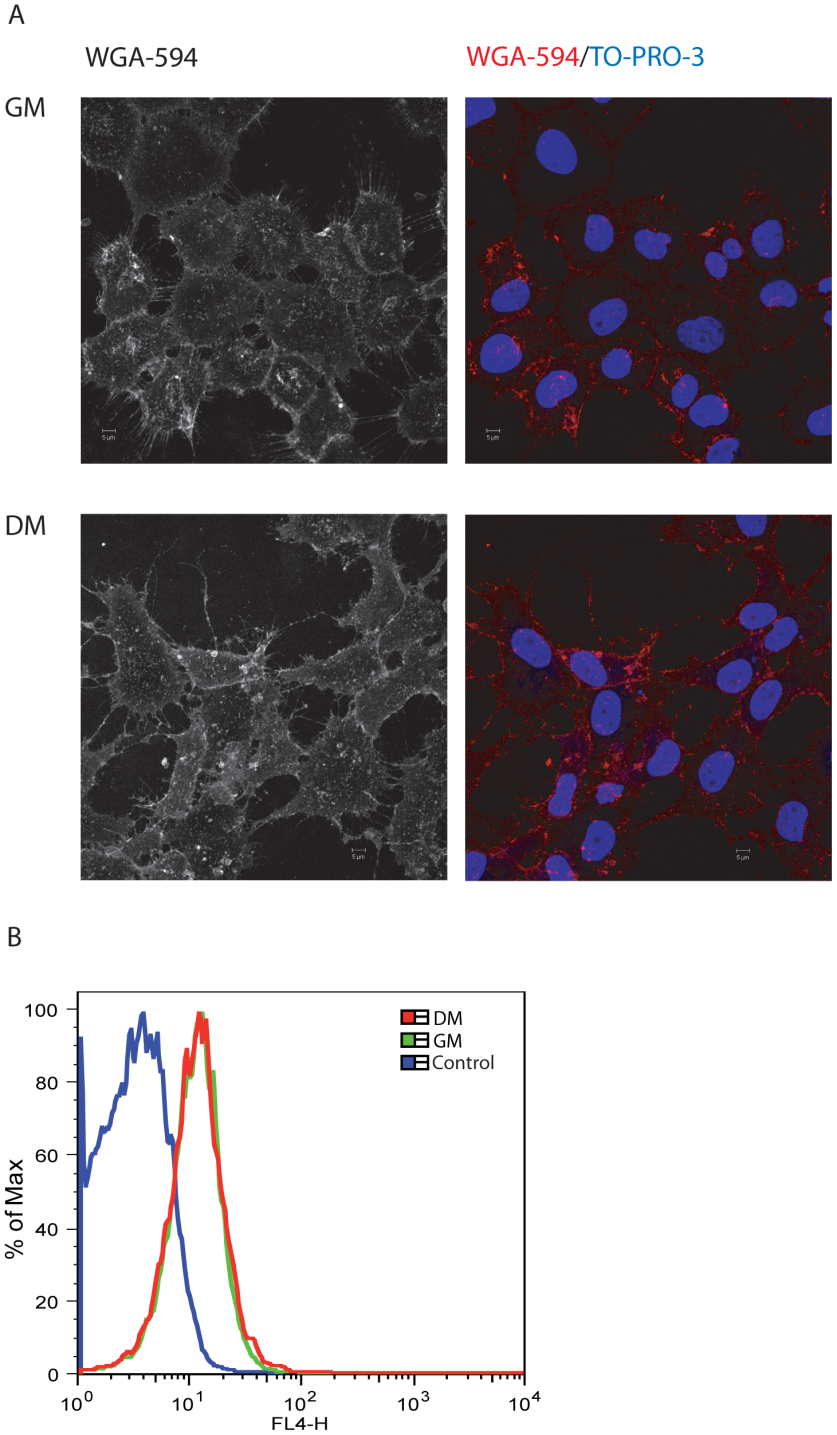
Expression of HSPGs in HOG cells. Cells previously cultured in GM or DM were incubated for 1 hour at 4°C with WGA-594 diluted in DMEM without serum. Cells were then washed with PBS, fixed and processed for immunofluorescence microscopy analysis (A) or FACS (B). A. Images show no significant changes between GM and DM cultures. Confocal images correspond to the projection of the planes obtained by confocal microscopy. Nuclei were stained with TO-PRO-3. B. Percentage (%) of max designates the number of cells relative to the maximum fraction. Control cells correspond to HOG cells incubated for 1 hour at 4°C in DMEM without WGA-594.

To investigate whether the major cell receptors for HSV-1 play a role in the increase of susceptibility of differentiated OLs to the infection, we monitored expression of HVEM, nectin-1 and 3-OS HS along the process of differentiation. We first analysed expression of HSPG, which act as an attachment factor for HSV gC and gB, by immunofluorescence assay. We incubated HOG cells in GM or DM with wheat germ agglutinin (WGA), a lectin that binds to N-acetylglucosamine, coupled to Alexa-594. HSPG was highly expressed on the surface of HOG cells and no significant changes were observed during differentiation (Figure 3A and B). In contrast, the detection of the specifically modified 3-OS-HS, which acts as a receptor for gD, with monoclonal antibody HS4C3 was negative in HOG cell line under growth and differentiation conditions (data not shown).

**Figure 4:**
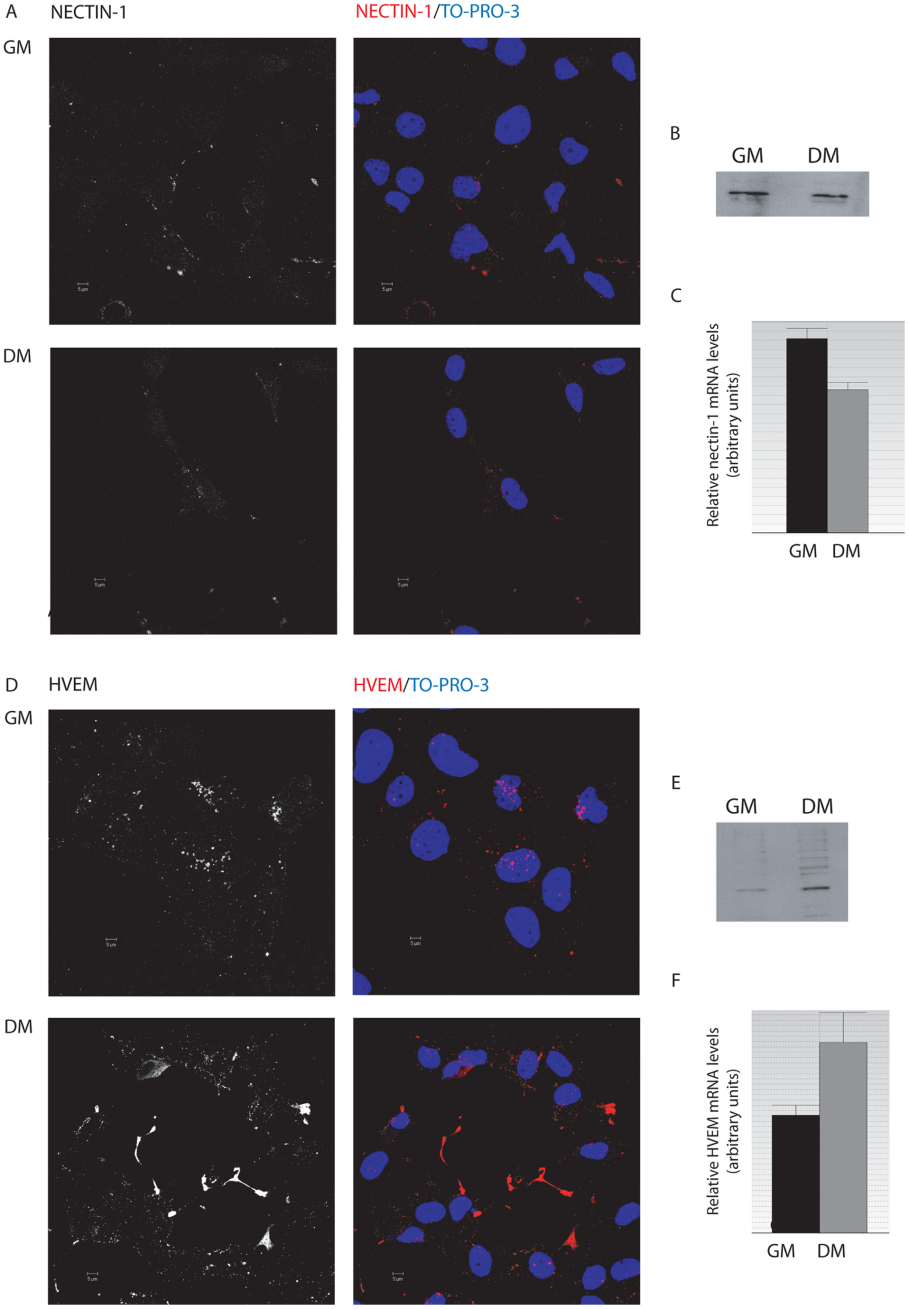
Expression of nectin-1 and HVEM in HOG cells. A. Cells cultured in GM or DM were fixed and processed for confocal immunofluorescence analysis with CK41 anti-nectin-1 antibody. Primary antibody was detected using an Alexa Fluor 555 secondary antibody. Images correspond to the projection of the planes obtained by confocal microscopy. B. HOG cells were subjected to SDS–PAGE and analyzed by immunoblotting with an anti-nectin-1 polyclonal antibody. Immunoblot assays showed a slight decrease of nectin-1 in cells cultured in DM. C. RTqPCR quantification of relative nectin-1 mRNA expression levels in HOG cells cultured in GM or DM showed a decrease in nectin-1 expression in differentiated cells compared to cells cultured in growth conditions. D. Cells cultured in GM or DM were fixed and processed for confocal immunofluorescence analysis with R140 anti-HVEM antibody. Primary antibody was detected using Alexa Fluor 555 secondary antibody. Images correspond to the projection of the planes obtained by confocal microscopy. E. HOG cells were subjected to SDS–PAGE and analyzed by immunoblotting with an anti-HVEM polyclonal antibody. Immunoblot assays showed an increase of HVEM in cells cultured in DM. C. RTqPCR quantification of relative HVEM mRNA expression levels in HOG cells cultured in GM or DM showed a significant increase in HVEM expression in differentiated cells compared to cells cultured in growth conditions. DIC: Differential Interference Contrast.

When we performed similar immunofluorescence assays with antibodies against nectin-1 (CK41) and HVEM (R140), slight changes in these two HSV-1 receptors took place between growth and differentiation conditions. This assay was performed incubating live cells with the antibodies in serum-free DMEM for 20 minutes at 4°C. After that, cells were fixed and processed for immunofluorescence analysis as described in the materials and methods section. Nectin-1 detection was slightly decreased in HOG cells cultured in DM whereas HVEM expression increased (Figure 4A and D). Although immunofluorescence provides important information on the location of receptors and allows a rough comparison of expression levels, it is not a robust quantitative measure. To address the quantitative effect, immunoblot analysis was performed with anti-nectin-1 (CK6) or HVEM (mouse monoclonal) antibodies and confirmed these immunofluorescence results (Figure 4B and E). We loaded either equal number of cells or equal amount of protein, obtaining similar results in both cases. Finally, to determine whether HVEM and nectin-1 expression was modified following cell differentiation, we quantified the mRNA using RT-qPCR in cells cultured either in GM or DM. Quantitative RT-PCR confirmed an increase of HVEM and a slight decrease in nectin-1 expression in HOG cells cultured under differentiation conditions when compared to GM cultured cells (Figure 4C and F).

### Cell Receptors Involved in HSV-1 Entry into HOG Cells

**Figure 5:**
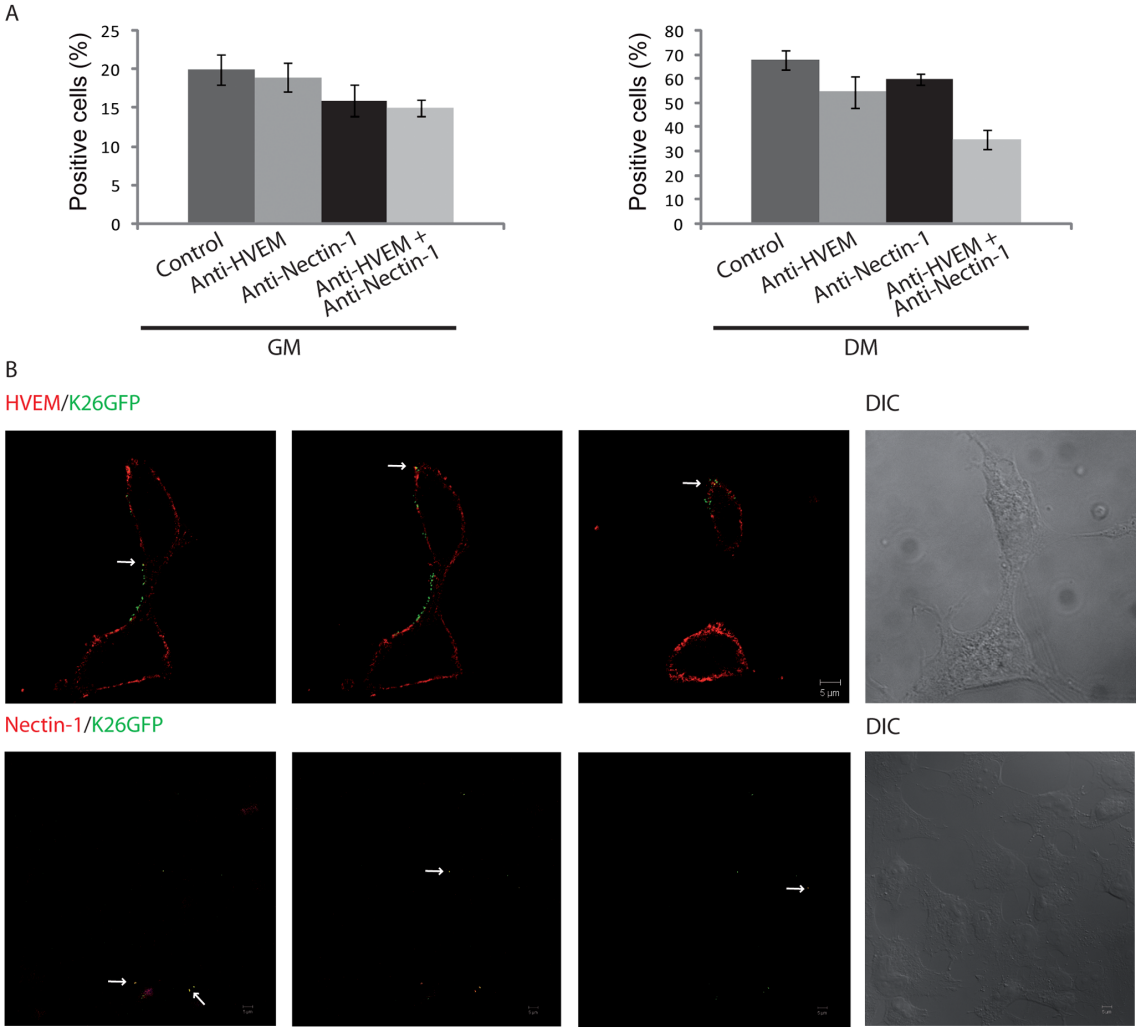
Role of HVEM and nectin-1 on viral entry in HOG cells. A. Antibody blocking assay was performed incubating HOG cells with R140 anti-HVEM or CK41 anti-nectin-1 or both antibodies simultaneously at 4°C for 1 h. Cells were then infected with an equal volume of K26GFP diluted in free serum medium at an m.o.i of 1 for 1 h at 4°C. After viral adsorption, cells were washed with PBS, incubated for 20 h with their respective media containing blocking antibodies and processed for flow cytometry. Controls correspond to cells not blocked with primary antibody. The percentage of infection in differentiated cells blocked with both antibodies simultaneously is lower than control without blocking antibodies (C). B. HOG cells cultured in DM and infected at an m.o.i. of 1 with K26GFP were fixed and processed for confocal indirect immunofluorescence analysis with R140 anti-HVEM and CK41 anti-nectin-1 monoclonal antibodies. Panels correspond to three confocal slices of 0.8 µm. Arrows point to colocalization of virus with receptors. DIC: Differential Interference Contrast.

Once the presence of nectin-1 and HVEM in the HOG cell surface was established, we analysed the role of these two receptors during the HSV-1 infection. We first carried out an antibody blocking assay. HOG cells incubated with anti-nectin-1 (CK41) or anti-HVEM (R140) or both antibodies simultaneously, were infected with K26GFP. After 20 h p.i, cells were fixed and processed for GFP flow cytometry as a measure of infection. Although we detected a slight blocking effect, especially with HVEM antibodies, neither anti-nectin-1 nor anti-HVEM antibody treatment efficiently blocked HSV-1 entry into HOG cells cultured in GM or in DM as compared to controls without blocking antibodies. Nevertheless, incubation with both antibodies simultaneously induced a more significant blocking effect (Figure 5A). On the other hand, analysis of HOG cells infected with K26GFP at 4°C for1h and processed for confocal indirect immunofluorescence analysis with anti-HVEM polyclonal and anti-nectin-1 monoclonal antibodies 5 minutes after the shift to 37°C, showed partial colocalization of viral particles with nectin-1 and HVEM (Figure 5B). These data suggest that both HVEM and nectin-1 are functional as HSV receptors in oligodendrocytic cells and that HVEM may play a bigger role when these cells differentiate.

### Study of Viral Entry by Electron Microscopy

**Figure 6:**
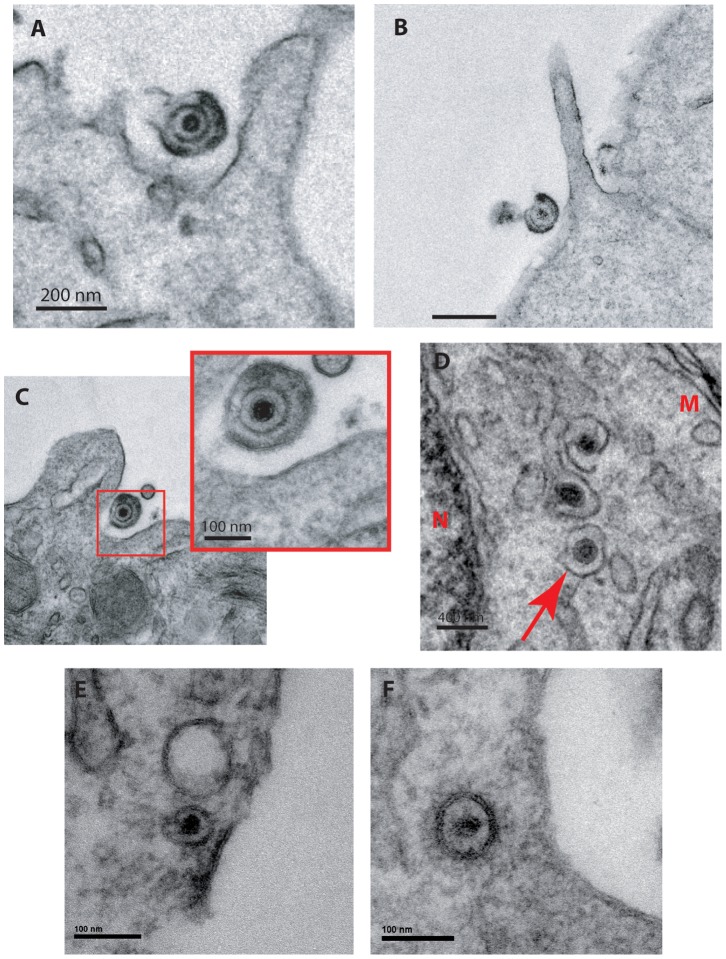
Study of viral entry by electron microscopy. HOG cells cultured in GM or DM were mock-infected or infected with HSV-1 at an m.o.i. of 50. At 5, 10, 20 and 30 minutes p.i., cells were fixed, washed and processed by freeze substitution. In cells cultured in DM membrane protrusions similar to planar lamellipodia (A and B) and circular ruffles (C) were observed at 20 minutes p.i., suggesting viral entry by macropinocytosis. Vesicles containing intracellular enveloped virions (D) (arrows) were also observed in cells cultured in DM at 30 minutes p.i., suggesting entry by endocytosis. Intracellular unenveloped virions were observed in cells cultured in GM and DM at 5 minutes p.i. (E and F), suggesting entry by fusion. Samples were examined with a JEM 1010 transmission EM (Jeol, Tokyo, Japan). N = nucleus. M = plasma membrane.

It has been proposed that HSV-1 entry can proceed by macropinocytosis/endocytosis in a cell-type dependent manner, regardless of which receptor in used [36], [56], [57], [58]. To determine whether endocytosis is involved in HSV entry into HOG cells, we used direct observation by electron microscopy. HOG cells cultured in GM or DM were mock-infected or infected with HSV-1 at an m.o.i. of 50. At 5, 10, 20 and 30 minutes p.i., cells were fixed and processed for observation (see material and methods). In cells cultured in DM for 20 minutes, membrane protrusions similar to planar lamellipodia (Figure 6A and B) and circular ruffles (Figure 6C) were observed in the vicinity of virions. These structures have been described as a part of the process of viral entry by macropinocytosis in different cells [58]. Furthermore, enveloped virions were detected in vesicles at 30 min post infection (Figure 6D), which is indicative of virus endocytosis. Altogether, these data suggest that macropinocytosis may be involved in HSV-1 entry into differentiated HOG cells. Finally, intracellular unenveloped virions were observed in cells cultured both in GM (Figure 6E) and DM (F) 5 minutes p.i., suggesting that membrane fusion had occurred. Thus, this pathway does not seem to be altered during differentiation.

## Discussion

HSV-1 can infect a diverse range of cell types and tissues including, neurons, retinal or conjunctival epithelial cells the as well as oral and genital mucosa [31], [32], [59], [60]. Previous studies have shown that OLs are highly susceptible to HSV-1 infection [13] and glial specific cellular proteins such as myelin-associated glycoprotein, MAG, have been implicated in viral entry [25]. In the present work, the effect of oligodendroglial cell differentiation on HSV-1 infection has been investigated. HOG cells exhibit characteristics related to immature OLs such as GalC and CNPase expression. Culturing of HOG cells in DM induces some differentiation hallmarks, such as proliferation of processes and the increase in MBP and MOG expression. Nevertheless it is not possible to find significant quantitative differences in GalC and CNPase between HOG cells grown in GM *versus* DM [61]. In a previous study [53] we observed the presence of myelin-like membrane sheets –structures previously described in primary and mixed cultures [62], [63], [64]– in HOG cells cultured in DM. We also detected an increment in PLP expression during differentiation of these HOG cells and accumulations of PLP in myelin-like sheets [55]. Moreover, these myelin-like sheets contained vesicles enriched in the apical molecule CD59 and MAL, a major myelin protein [53]. In summary, HOG cells cultured in DM acquire a more differentiated phenotype characterized by morphological features –elongation of processes and emergence of myelin-like sheets–, biochemical changes –an increase in MBP, MOG and PLP– and accumulation of myelin proteins –such as PLP and MAL– in myelin-like sheets. These characteristics make HOG cells an appropriate model to study changes in HSV-1 infection between cells with different developmental stages.

In this study, our results show that culturing HOG cells in DM or maintaining OPCs in differentiation culture conditions for 3 days enhanced HSV-1 infection. To ascertain possible factors involved in this increased susceptibility, we first monitored expression of the best characterized HSV-1 receptors (i.e. HVEM, nectin-1 and 3-OS-HS) along the process of HOG cell differentiation. Immunofluorescence microscopy revealed that expression of the attachment HSPGs remained elevated and unchanged throughout differentiation. In contrast, the specifically sulfated 3-OS HS was not detected in HOG cells using antibody HS4C3.

Furthermore, by means of immunofluorescence microscopy, immunoblot analysis and RT-qPCR, we have detected an increase of HVEM and a slight decrease of nectin-1 in HOG cells cultured in DM in comparison to GM treated cells. Previous works have demonstrated that nectin-1 has a major role in HSV-1 entry into neurons [33], [65]. In our oligodendroglial model, the expression of this receptor in HOG cells is rather low, but we observed some colocalization with viral particles. HVEM expression in HOG cells was higher than nectin-1, and, again, we observed colocalization of HVEM with viral particles. These results suggest that both nectin-1 and HVEM are functioning as HSV-1 receptors in HOG cells. Accordingly, blocking with either anti-nectin-1or anti-HVEM antibodies did not induce a significant decrease in viral infection since preventing the use of one receptor may lead the virus to take advantage of the other more extensively. In addition, blocking with both antibodies simultaneously induced a decrease in viral infection in differentiated cells, supporting the functional role of these receptors in viral entry into HOG cells depending on the differentiation stage. The fact that the combined effect is greater in differentiated HOG cells, where HVEM is more highly expressed, suggest that a basal level of nectin-1 activity is present in all cells. However in differentiated cells the anti-HVEM antibody-alone and in combination with anti-nectin-1 is more potent, thereby highlighting the predominant role of HVEM in differentiated cells. It is noticeable that under our experimental conditions, anti-HVEM and anti-nectin-1 antibodies together did not completely block entry. In other systems, 3-OS HS is the major receptor for HSV-1 [66]. Because nectin-1 and HVEM are not the only receptors for HSV, we attempted to address the role of 3-OS-HS as an entry receptor in these cells. In preliminary experiments, we were unable to specifically detect 3-OS-HS in HOG cells using antibody HS4C3. However, these data do not allow us to rule out a role for 3-OS-HS in HSV-1 entry into oligodendrocytes. Such an activity could partly account for the residual entry observed in the presence of antibodies blocking nectin-1 and HVEM. 3-OS-HS are generated by six isoforms of HS 3-O-STs. At the moment, there are no available data about the set of 3-O-STs expressed in human oligodendrocytes. Expression of 2-O-ST, the enzyme responsible for 2-O-sulfation, is downregulated during maturation of OLs, although an increase in 2-OS-HS has been observed after injury to the adult rat brain [67]. 3-O-STs are often co-expressed in various combinations. While the isoforms 3-O-ST-3, −5 and −6 are most commonly expressed, isoforms 3- O-ST-2 and −4 were undetectable in other cell lines examined [35]. This complexity warrants further experiments to determine the expression pattern of 3-OS-STs during oligodendrocyte differentiation and determine whether 3-OS-HS plays a role in HSV entry in these cells.

MAG is a cell-surface molecule expressed in myelin sheath [68], [69]. MAG is involved in myelin maintenance and in myelin-axon interactions, acting as an inhibitor of axonal regeneration [70], [71] It has been reported that MAG is associated with HSV-1 gB, suggesting that it is involved in HSV infection of neural tissues [25]. In our cellular model, preliminary studies by RTqPCR revealed a negligible expression of this myelin protein in HOG cells even in differentiation culture conditions (data not shown), thus suggesting that the role of this protein in viral entry into HOG cells is very limited at best.

HSV-1 can enter different cell types using different pathways: fusion at a neutral pH, low-pH-dependent endocytosis and low-pH-independent endocytosis [56], [57], [72]. We used electron microscopy to define the entry pathways of HSV-1 into OLs. This approach is useful to directly observe virions at various stages of entry. However, interpretation of EM snapshots needs to be related with functional data to validate the fact that observed virions reflect a functional entry pathway. In cells cultured in DM membrane protrusions similar to planar lamellipodia and circular ruffles [73] were observed, suggesting that HSV-1 may be entering OLs by macropinocytosis (depending on their differentiation stage). Similar cellular protrusions have been associated to HSV-1 during entry by phagocytosis-like uptake involving re-arrangement of actin cytoskeleton and trafficking of the viral particles in phagosome-like vesicles. This pH-dependent and clathrin-independent viral entry is characterized by the presence of cell surface protrusions and clustering of gD receptors in large vesicles [58]. In addition, vesicles containing intracellular enveloped virions were also observed in cells cultured in DM, suggesting entry by endocytosis. Further studies will be necessary to define the role of pH in this pathway.

Finally, viruses like HBV, HPV and HIV have been shown to induce cell differentiation [74], [75], [76], [77]. Therefore, we wanted to ascertain not only whether differentiation triggered changes in HOG cell susceptibility to HSV-1, but also whether HSV-1 was able to drive changes in cell morphology compatible with cell differentiation. After infection, immunofluorescence microscopy revealed an increase of PLP in cells infected with HSV-1 cultured in growth conditions. Also, morphological changes corresponding to differentiated cells were also observed in infected cells cultured in GM. Moreover, HSV-1 infected MAL-expressing HOG cells cultured in GM exhibited myelin-like sheets enriched in exogenous MAL. Altogether, these observations indicate that HSV-1 infection can induce the formation of structures corresponding to more differentiated stages of oligodendrocytes. Unexpectedly, partial colocalization between HSV-1 and exogenous MAL was detected, but the significance of that observation remains unclear. Colocalization of HSV-1 and exogenous MAL appears most prominently in vesicles located at the end of the processes. This raises the possibility that MAL-positive vesicles may be involved in delivering viral particles towards the end of the processes and subsequently, outside the cells. However, further studies will be necessary to specifically address the potential role of MAL in HSV-1 egress.
